# Learning Through a Pandemic: Youth Experiences With Remote Learning During the COVID-19 Pandemic

**DOI:** 10.1177/21582440221124122

**Published:** 2022-09-24

**Authors:** Nadia Nandlall, Lisa D. Hawke, Em Hayes, Karleigh Darnay, Mardi Daley, Jacqueline Relihan, Joanna Henderson

**Affiliations:** 1 Margaret and Wallace McCain Centre for Child, Youth and Family Mental Health, Centre for Addiction and Mental Health, Toronto, ON, Canada; 2 University of Toronto, ON, Canada

**Keywords:** adolescent, COVID-19, education, mental health, virtual learning, youth

## Abstract

The objective of this paper was to examine the school-related experiences of youth during the COVID-19 pandemic. Participants represented both clinical and community youth aged 14 to 28 who were sampled as part of a larger study. Feedback from youth attending school during the pandemic was qualitatively examined and youth who planned to attend school prior to the pandemic and did (*n* = 246) and youth who planned to attend but did not (*n* = 28) were compared quantitatively. Youth appreciated the flexibility of online learning and some also reported experiencing a lack of support from their school and the need for instructor training on how to deliver virtual classes effectively. Future studies should examine what factors influence student engagement with virtual learning, what strategies could improve supports for student in their long-term career development, and the longitudinal experiences of youth who may have chosen not to go back to school due to the pandemic. This survey was conducted in Ontario, Canada. A more diverse sample collected outside of Ontario would improve generalizability. Qualitative data were based on survey responses and not interviews. Thus we were unable to discern the reasons youth decided to attend school, or not, during the COVID-19 pandemic.

The COVID-19 pandemic has caused major disruptions to educational systems around the world (United Nations, 2020). Social distancing measures have required school closures, shifting in-person learning to remote learning or a hybrid format that combines remote and in-person formats. To comply with these measures, youth have had to substantially adapt their routines, activities, and plans. Some youth are now attending school online, have limited access to leisure activities and many rely on technology as a means of communication with friends, peers, and teachers. As a consequence, many youth are unable to engage in social interactions with friends, peers or teachers in ways that are familiar to them. Disruptions to school and social relationships present a unique challenge for youth who, at a critical stage in their social development, are at risk for mental health challenges (Merikangas et al., 2010; Rogers et al., 2021) and rely on peer connections for social and emotional support (Pigaiani et al., 2020; Steinberg & Morris, 2001). Some studies have quantified student experience with remote learning during the pandemic (Godoy et al., 2021; Horita et al., 2021; Lischer et al., 2021; Meda et al., 2021), however, there is limited qualitative feedback about this experience from students while attending school. It is imperative that we understand student experience with remote learning as we navigate educational priorities in a post-pandemic world.

Early adulthood is characterized by ongoing cognitive, social and emotional changes (Azzopardi et al., 2019; Patton et al., 2016). This is an important period underscored by several developmental tasks including independence, progress through the educational system, transition to employment, civic engagement, and the development of relationships (Patton et al., 2016). This is also a time when social connectedness and social identity are salient to early adulthood development (Matthews et al., 2019; Pigaiani et al., 2020; Power et al., 2020; Steinberg & Morris, 2001). During this period, attachment and social belonging needs are sought through friendships as youth strive to gain independence from their parents (Meuwese et al., 2017; Nickerson & Nagle, 2005; Steinberg & Morris, 2001). In this respect, the school environment plays an important role, because it is a place where youth seek to establish peer relationships and develop a social identity (Bailen et al., 2019; Nickerson & Nagle, 2005; Scott et al., 2021; Steinberg & Morris, 2001). Since opportunities for social activities like in-person interaction with teachers and classroom socialization have been disrupted due to the COVID-19 pandemic, youth are participating in fewer activities that are necessary for their optimal growth and development (Adnan, 2020). In fact, youth report a loss of emotional connection and friend support during the pandemic even despite their ability to use technology to interact (Rogers et al., 2021). The reported decline in friend support during the pandemic is associated with more depressive symptoms, conflict with friends and loneliness (Rogers et al., 2021). This is particularly problematic as youth typically experience feelings of loneliness (Matthews et al., 2019). Given the important role that the school environment plays in providing social support, the disruption to daily social interaction associated with the pandemic’s impact on learning institutions is of consequence to this population in the context of their developmental needs during this period (Patton et al., 2016; Power et al., 2020).

Mental health disorders are likely to first emerge during adolescence and can extend into adulthood (Allen et al., 2008; Bailen et al., 2019; Merikangas et al., 2010). Onset of depression, anxiety and suicidality have been associated with the frequency of fluctuating and intense emotions experienced by youth (Allen et al., 2008; Merikangas et al., 2010). In Canada, approximately one in five youth had mental health and/or substance use challenges prior to the COVID-19 pandemic (Wiens et al., 2020). Youth are also experiencing mental health or substance use challenges during the pandemic (Hawke et al., 2020, 2021; Liang et al., 2020; Shanahan et al., 2022; Son et al., 2020) and that the disruption to school routines and self-isolation are associated with increased mental health challenges (Jiao et al., 2020; Singh et al., 2020). This is problematic as students with mental health needs are at risk of poor academic outcomes due to their higher levels of disengagement and early school leaving (Arria et al., 2013; Eisenberg et al., 2009; Lipson & Eisenberg, 2018). Even though mental health challenges among post-secondary students were on the rise pre-pandemic (Blanco et al., 2008; Dalky & Gharaibeh, 2019; Wiens et al., 2020), students are reporting higher rates of depressive and suicidal thoughts during the COVID-19 pandemic than pre-pandemic (Basheti et al., 2021; Son et al., 2020). This is consistent with an earlier study in which we found significant deterioration of mental health among youth in both clinical and community samples during the early stages of the pandemic (Hawke et al., 2021). Another study examining the psychological distress of university students during the COVID-19 pandemic found that students without pre-existing mental health needs were more likely to show an increased risk of mental health needs compared to students who reported pre-existing mental health needs 1 year prior to the pandemic (Hamza et al., 2021). Studies have also demonstrated the deteriorating effects of lockdown restrictions; approximately 6% of students experienced depressive symptoms and disruptions to sleeping habits, daily fitness routines, and social interaction significantly affected their health during this period (Chaturvedi et al., 2021; Meda et al., 2021).

With a lens to stakeholder engagement, the aim of the Margaret and Wallace McCain Centre for Child, Youth and Family Mental Health, at the Centre for Addiction and Mental Health in Toronto, Canada, is to advance research that is reflective of the preferences and needs of youth and their caregivers and integrate this knowledge into system delivery (Heffernan et al., 2017; Henderson et al., 2013). We engaged our youth partners in the development of our survey questions. Our youth partners identified going back to school during the pandemic as a salient concern among youth. Specifically, they wanted to learn about the experiences of youth attending school during the pandemic, how well educational institutions were handling the pandemic and how youth were adjusting to remote learning in the context of school.

Given the uncertainties imposed by the COVID-19 pandemic, governments and educational institutions are at a crossroads when it comes to understanding the impacts of the pandemic and its implications for future resource planning. As such, it is important for educational institutions to receive feedback from youth of their experiences with school during the COVID-19 pandemic. This knowledge could not only help improve the delivery of academic programs, it also has implications for post-pandemic planning to support student wellbeing (Hinderaker, 2013). This study helps to address this issue by surveying students recruited from clinical and community samples about their experiences of going back to school during the COVID-19 pandemic.

## Objective

This paper examines the school-related experiences of youth during the COVID-19 pandemic. Quantitatively, we examine youth who attended school during the pandemic and those who planned to attend school but did not. Qualitative data on youth experiences with attending school during the pandemic, school system handling of the pandemic and experience with digital video platforms are also analyzed.

## Method

This is a multi-methods study in which both qualitative and quantitative data were collected simultaneously and analyzed in parallel (Fink, 1993). Data for this study were collected as part of a broader mixed methods longitudinal study of youth experiences in the context of the COVID-19 pandemic (Hawke et al., 2021). Specifically, this paper analyzes the October 2020 wave of data collection. The phases of study data collection are as follows: baseline demographic characteristics were collected in April 2020 as part of the broader longitudinal mixed-methods study; data on psychiatric and behavioral health disorders were collected in June 2020; and education-related experiences and current overall self-rated mental health were collected in October 2020. Youth were defined as young people aged 14 to 28 (Statistics Canada, 2019b) at the October 2020 wave of data collection.

### Participants

The full longitudinal study sample consisted of 622 participants. The current study reports on a subsample of those participants who had planned to attend school in September 2020 (the new academic year) in our October wave of data collection (*n* = 419). Two groups were created from our subsample of youth who planned to attend to school prior to the pandemic for the new academic year in September 2020: those who planned to attend school prior to the pandemic and did attend school during the pandemic and (*n* = 246) and those who planned to attend school prior to the pandemic but did not attend (*n* = 28). Participants were between the ages of 14 and 28 (*M* = 20.2, *SD* = 2.2), among whom 184 (67%) reported girl/woman gender identity and 10 (3%) youth whom reported transgender or gender diverse identity. Details on the full sample are described in Hawke et al. (2020).

### Procedure

Participants received an email message with a web link taking them to an online survey on REDCap software (Harris et al., 2009). After informed consent, youth completed a 20 to 30 minute survey about their experiences during the COVID-19 pandemic. Data collection took place between October 6 and October 29, 2020, approximately 8 months after the COVID-19 pandemic as declared by the World Health Organization (2020) and a state of emergency was declared provincially (Government of Ontario, 2020) and starting a month after the 2020–2021 academic year began locally (i.e., early September). Centre for Addiction and Mental Health Research Ethics Board approval was obtained.

### Measures

Demographics: Demographic characteristics were collected including age, gender identity, ethnic background, education status, employment status, and other variables.

The Youth Self-Report Baseline version 0.1 of the National Institute of Mental Health (NIMH)-developed CoRonavIruS Health Impact Survey (CRISIS) tool was used to examine COVID-19 impacts on participants aligning with the NIH COVID-19 research priorities (Holmes et al., 2020). The measure examines emotional and behavioral responses retrospectively at 3 months prior to the COVID-19 crisis and in the past 2 weeks, as well as service use, service disruption, and other constructs (Hawke et al., 2020).

Psychiatric and behavioral health disorders: The GAIN-Short Screener (GAIN-SS) is a self-administered screener to identify the extent to which participants are experiencing symptoms of one or more mental or behavioral health disorders (e.g., internalizing or externalizing psychiatric disorders, problematic substance use or problems with crime and violence) (Dennis et al., 2006). Responses are given in relation to how recently the participant experienced the described problem. The scale ranges from 0 (never) to 4 (past month). Three or more items endorsed per subscale as present in the past-month suggest a high likelihood of meeting diagnostic criteria during the past month (Dennis et al., 2006, 2008), including among youth (Dennis et al., 2006). The GAIN-SS scores reported are from the June 2020 wave of data collection, prior to the start of the school year in September 2020, in order to examine whether pre-academic term mental health was associated with choosing to attend school.

Overall mental health: Youth were also asked in October 2020 to provide a self-rating of their mental health on a five-point scale ranging from poor to excellent, aligning with a question typically asked by Statistics Canada (Statistics Canada, 2019a).

Education survey: Aligning with the McCain Model of Youth Engagement (Heffernan et al., 2017), survey questions were developed by the research team, including youth co-researchers. Questions were developed to understand education-related experiences during the pandemic and were embedded as part of the larger survey. Participants who identified as attending school during the pandemic were asked to provide qualitative comments about their school experiences. The specific survey questions were: if you’re a student, how has back to school gone during the pandemic?; how well is the school system handling the pandemic and how could they do better?; during the pandemic, you may have been spending a lot of time on platforms like Zoom and Houseparty. How is this going for you? How do you feel about having to be on camera so often? While the latter questions were not specific to school, youth reported their experience with using these platforms for remote learning. Participants who reported on planning to attend school were categorized by school level as reported in April 2020: those who had completed grade 11 or more as of April 2020 (i.e., were in grade 12 in April 2020) were categorized as post-secondary students (*N* = 253) and those completed grade 10 or less in April 2020 were categorized as secondary students (*N* = 21).

### Analyses

Frequencies were calculated to describe the sample. Frequency is a measure of the number of occurrences of a particular score in a given set of data and it is a statistical method typically used to help characterize the population (Mihaescu, 2010). Chi-square tests were used to determine whether differences between school attendees and non-attendees were statistically significant; non-parametric tests were used to accommodate unequal sample sizes because this test does not rely on assumptions that the data are drawn from a specific distribution (Mihaescu, 2010). For the purposes of this study, GAIN-SS domains for internalizing, externalizing and problematic substance use were analyzed; the crime/violence scale was not analyzed due to low endorsement rates. Effect sizes were calculated as phi (φ) coefficients, Cramer’s V, or Fishers Exact test as appropriate.

A qualitative thematic analysis was conducted on each open-ended question to collect participants’ feedback on different aspects of attending school and using virtual platforms during the COVID-19 pandemic consistent with criteria for trustworthiness in qualitative research (Nowell et al., 2017) and methodology for thematic analyses as described by Braun and Clarke (2006). Thematic analysis was chosen because it is a highly flexible approach that can provide a rich and detailed account of complex data (Nowell et al., 2017). Qualitative comments were uploaded verbatim into Nvivo software (Bazeley, 2013). Next, all comments were read through once before the researcher began coding to ensure familiarity with the data. Initial coding of the data was then conducted to identify aspects of the data which were relevant to the research questions. These codes helped to organize the content of the data around the research questions. Next, themes were developed in relation to each code. Themes and sub-themes were reread and were either combined if they were similar or eliminated based on their frequency (e.g., if a theme appeared less than two times it was eliminated to ensure accurate representation of the data). This was an iterative process. Themes were then further refined through review with a co-author (LDH). After this process, the themes were finalized. Data were analyzed separately for participants who reported attending school virtually versus in-person. However, the themes identified were consistent across groups, with those attending in-person also reported on the challenges associated with virtual learning. Thus, combined qualitative results are presented. Qualitative data were also examined by gender and level of education (i.e., secondary vs. post-secondary levels) but themes were consistent across groups and thus, combined results are presented across genders and levels of education.

## Results

Table 1 presents demographic and self-reported school, employment and mental health-related characteristics of participants from the two groups created from our subsample of participants who planned to attend to school prior to the pandemic for the new academic year in September 2020: those who planned to attend school prior to the pandemic and did attend school during the pandemic and (*n* = 246) and those who planned to attend school prior to the pandemic but did not attend (*n* = 28). These two groups were then compared.

**Table 1.** Demographic and Self-Reported Health Characteristics of Participants by Sample.

		Total study sample (*n* = 274)	Planned for school but did not go (*n* = 28)		Went to school (*n* = 246)		*p*	Phi/Cramer’s V
			*N*	%	*N*	%		
Age	Less than 18 years old	26	4	15.3	22	84.6	.282	0.097
	Between 18 and 22 years old	215	19	8.8	196	91.2		
	Between 23 and 28 years old	30	5	16.7	25	83.3		
Gender^1^	Man/boy	80	13	16.2	67	83.8	.050	0.121
	Woman/girl	184	15	8.2	169	91.8		
	Transgender or non-binary	10	0	0.0	10	100		
Ethnic origin/background^2^	Caucasian	153	16	10.5	137	89.5	.605	0.082
	Asian	63	5	7.9	58	92.1		
	Mixed heritage	25	4	16.0	21	84.0		
	Another background	32	2	6.2	30	93.8		
First language English		246	24	9.8	222	90.2	.453	0.045
Born in Canada		239	25	10.5	214	89.5	.730	−.021
Geographical location	Large town/suburbs	197	20	10.1	177	89.9	.954	0.004
	Small town/rural	77	8	10.4	69	89.6		
Highest level of education completed as of April 2020	Up to grade 10 (expected secondary level in Oct. 2020)	21	3	14.3	18	85.7	.522	0.410
	Grade 11 or higher (expected post-secondary in Oct. 2020)	253	25	9.9	228	90.1		
Living situation^3^	Live with parents/family home	185	19	10.3	166	89.7	.735	0.021
	Live independently	77	9	11.7	68	88.3		
Student status	Full time	196	n/a	n/a	196	100	n/a	
	Part time	46	n/a	n/a	46	100		
	Other	4	n/a	n/a	4	100		
Mode of learning	In-person	57	n/a	n/a	57	100	n/a	
	Virtual	185	n/a	n/a	185	100		
Employment status^4^	Full time	22	4	18.2	18	81.8	.337	0.111
	Part time	35	4	11.4	31	88.6		
	Unemployed	199	20	10.1	179	89.9		
	Other (e.g., internship, volunteer, etc.)	16	0	0.0	16	100		
Self-reported physical/social distancing from others	Usually/always	253	23	9.1	230	90.9	.116	−.095
	Rarely/never	20	4	20.0	16	80.0		
Self-rated current mental health status	Excellent/good	134	9	6.7	125	93.3	.058	3.583
	Fair/poor	139	19	13.7	120	86.3		
Mental health and substance use^5^	GAIN-SS Internalizing	118	13	11.0	105	89.0	.767	0.020
	GAIN-SS Externalizing	45	4	8.9	41	91.1	.705	−.025
	GAIN-SS Substance use	19	2	10.5	17	89.5	.989	0.001

Significance tests conducted on the following groups: ^1^man/boy versus woman/girl; ^2^Caucasian versus other background; ^3^consistent housing versus precarious housing; ^4^employed versus unemployed. ^5^Fishers Exact Test results are reported to accommodate for small cell sizes.

Results show that one in ten youth (10.2%) who planned to go to school did not. Seventy-five percent of youth who reported attending school attended virtually and 23.3% of participants reported attending school in-person. However, participants who reported attending school in-person also provided qualitative comments on their experience with virtual learning, likely reflecting the use of hybrid models and/or intermittent use of remote learning during emergency lock-down stages.

Chi-square tests conducted for most demographic and clinical characteristics revealed no significant differences between those who attended school during the pandemic and those who planned to attend but did not (*p*s > .05, Table 1). However, there was a trend toward significance for gender (man/boy vs. woman/girl) χ^2^ (1, *N* = 264) = 3.856, *p* = .050, φ = 0.121, with a small effect size suggesting that more men/boys may have decided not to attend school compared to woman/girls. There was also a trend for current self-reported mental health status (1-item), χ^2^ (1, *N* = 273) = 3.583, *p* = .058, φ = 0.115, with a small effect size suggesting that those who planned but did not go to school may have had worse mental health status as reported in June 2020 than those who did attend school.

### Qualitative Findings

Among participants who did attend school, the qualitative findings reveal a mix of positive and negative experiences with attending school during the pandemic, with the vast majority of qualitative responses focused on experiences of virtual learning. Some participants who attended school during the pandemic reported that they enjoyed the flexibility of remote learning. Lack of clear and consistent communication and increased emphasis on self-directed learning were reported as challenges to remote learning, while participants suggested that reducing school workload would improve the school experience. Most participants reported feeling comfortable using digital platforms, but many preferred to turn off their cameras and not be seen on video during their classes. The physical effects of increased screen time and the financial burden of high tuition costs without the full school experience are ongoing concerns.

#### Positive experiences with attending school during the pandemic

The themes related to positive experiences with attending school include: flexibility in program delivery, student adaptability, and instructor characteristics.

##### Flexibility in program delivery

Participants appreciated the flexibility of remote learning, offering them the ability to set their own schedule, work at their own pace and have more free time:

*“I had problems attending classes, but with courses online and no set schedule, I feel I can manage the workload a lot better and in general improve my focus, while reducing my stress.”*

*“Remote learning and classes gave me more time to pursue interests outside of school.”*

##### Student adaptability

After a period of adjustment, student ability to adapt to the new format helped them feel more positive about virtual learning:

*“It is a different experience for sure. Trying to adapt to the online learning aspect has been a learning curve, but now I am used to it and have come to enjoy it.”*

“*I already got a taste of remote learning through my spring 2020 term, where I had trouble adjusting to everything (not being able to be on campus, adjusting to spending more hours on my laptop minus social in-person interaction with my friends, and test-taking). This term has gone smoother because I had a better idea of what to expect from online learning and I also knew how to better study and prepare for evaluations. . .”*

##### Instructor characteristics

A few participants qualitatively reported that instructor support in terms of clear and consistent communication, instructor availability and capability for delivering online classes improved their school experience:

*“My professors are very supportive, providing clear guidelines, and even doing check in phone calls with us individually.”*

*“My professors are decent, and have done well acclimating to teaching online, which is good.”*

#### Negative experiences with attending school during the pandemic

The themes related to negative experiences with attending school during the pandemic included: challenges with self-management, isolation, emphasis on self-directed learning, limited learning opportunities, lack of engaging program material/delivery, workload and lack of instructor skills in delivering virtual classes.

##### Challenges with self-management

Some participants reported challenges with self-motivation, self-directed learning, time-management, accountability and concentration.

*“Getting used to the online environment has been very stressful. I have difficulty with time management and I have difficulty staying motivated.”*

*“I have had a lot of trouble concentrating on school as my classes are not live and just posted. I have trouble finding the motivation to do school from home. It would be easier for me to go in person.”*

##### Isolation

As a consequence of remote learning, participants reported that feelings of loneliness and the inability to meet new people contributed to feelings of isolation.

*“It’s my first year of university and I’ve moved across the country alone; feeling very isolated from others, unsure of how to connect with those in my community and feeling very distant from my friends from back home.”*

*“This has become extremely exhausting, isolating, and numbing–hence why I dropped from full-time to part-time status.”*

##### Emphasis on self-directed learning

The emphasis on self-directed learning by instructors has increased stress for some participants. This was problematic for some participants who were in first-year university or college, science-based courses, and/or those who expressed challenges with self-management (e.g., self-motivation, time-management and concentration):

*“This is my first semester of college ever so it’s an adjustment no matter what. But I hate being in online school, I have a hard time staying on top of things and we have a LOT of work to do on our own time I have a hard time staying on top of it.”*

*“It has been stressful trying to navigate the new format while receiving the same level of information that is supposed to be in person. We basically have to teach ourselves university-level science courses.”*

##### Limited learning opportunities

Participants reported limited learning opportunities because they are not able to take part in regular school activities such as labs and other hands-on learning and practicums, due to social distancing restrictions.

*“Only problem is the lost opportunity to work with lab equipment and perform hands-on learning activities.”*

*“It’s been quite stressful; since I am in nursing, which is a very hands-on major, I’ve found it difficult to learn different concepts.”*

##### Lack of engaging program material/delivery

Participants reported that program delivery was often monotonous, rigid and not adapted for online delivery; they suggested that instructors develop approaches to make program content and delivery more engaging.

*“A mix of stress and sheer boredom. As any year, there’s a lot of academic pressure (tests, deadlines, etc.), but all of it happens within one screen, sitting in the same chair, at the same desk.”*

*“I also despise the fact that these virtual courses are designed exactly the same way as in-person courses: Do these instructors not understand the toll of isolation and how exhausting it is to be in this situation in the first place? I feel that there must be adjustments made, and my classmates feel the same way.”*

##### Workload

The large number of assignments, often with short deadlines, was a source of great stress for participants as many qualitatively reported far more schoolwork compared to in-person learning:

*“Online schooling feels as if there is more emphasis on meeting deadlines rather than actually consuming the material and learning. I’m not sure if this is just to ready me for adult responsibilities or if the school is botching my learning experience.”*

*“Professors have taken advantage of things being online and are over-assigning work. They have overlapped multiple due dates and made time management very difficult. Lack of instructor skills in delivering virtual classes.’’*

*Participants reported frustration with instructors’ limited knowledge or ability for delivering classes online.*

*“I wish the professors were more engaging and didn’t sound like they just got out of bed.”*

*“School is very unorganized and teachers are uneducated about online and virtual classes.”*

*“. . .It’s awful, I hate online school and a lot of my professors are not tech savvy, so its been frustrating. . .”*

#### School system handling of the pandemic

Participants qualitatively reported both positive and negative perspectives on their schools’ handling of returning to school during the pandemic. Themes related to the school system handling of the pandemic include: insufficient communication, lack of support from instructors, need for improved student supports, reassured by COVID-19 prevention and control measures and the high cost of tuition.

##### Communication

Participants reported a need for frequent and consistent communication about school-related announcements; they felt that they could improve the school system’s response if they had opportunities to provide feedback to schools on their experience and ideas for improvement.

*“The school system should take action to have better communication with their students about announcements and schoolwork.”*

*“I think one thing the school could do better would be sending out more surveys to ask students how they think online learning is to continuously make improvements as it is unclear when we will return to in-person course instruction.”*

##### Lack of support from instructors

Participants reported that a lack of support from instructors, such as unavailability to answer questions, has negatively their remote school experience during the pandemic.

*“Force professors to answer questions promptly and without attitude or unhelpful responses like ‘read the rubric.’”*

*“Computer science is really awful because the professor has burnout and is still assigning a lot of assignments and not helping us when we have questions.”*

##### Student support

Some participants reported that they are not receiving the support they need from their school with respect to technology, financial resources, mental health, and teacher engagement.

*“Providing supports for students with relief money, support with technology. They could do better by creating standards for professors to follow (ex. ensuring professors can’t lecture longer than the allocated class time, ensuring that class work is proportionate to previous years, and ensure that profs are making themselves available for questions).”*

*“The system can also offer more support to students with regards to financial aid and mental health support.”*

##### Reassured by COVID-19 prevention and control measures

Participants also expressed gratitude for the safety measures put in place by the school protecting them from COVID-19:

*“So far so good. Online lectures aren’t ideal but I’m very thankful they are an option. I would much prefer an online lecture than COVID, so overall I’m happy.”*

*“They are making the best of a bad situation. I don’t think they could make it any safer if they opened the campus up. I prefer to stay home.”*

##### Tuition

Participants felt that tuition should be adjusted to account for the change from in-class to remote learning and reduced use of physical resources.

*“I don’t understand why I struggled to pay over 7k for tuition to only watch pre-recorded monotone videos and* [slide presentation program]*”*

*“I’m paying tuition to learn from [video platform] videos in my own home and I went to school away from my home city specifically to escape the negative energy in my home – and now I’m stuck here.”*

#### Experience with digital video platforms

A large proportion of participants qualitatively reported that they felt comfortable using digital platforms for school, but often turned off their camera because it made them feel awkward, anxious, or self-conscious. On the other hand, some participants reported that they liked video conferencing because seeing other people gave them a sense of community and feelings of support. Themes related to the experience with digital video platforms include: uncomfortable on camera, human connection via technology, disruptive to learning, physical complaints, and security/privacy issues.

##### Uncomfortable on camera

Participants reported that they were comfortable using video technology but that being on camera for school made them feel awkward, anxious, insecure and self-conscious. However, some participants reported that while they were not comfortable being on camera for school, they were comfortable using this technology with family and friends.

*“I do not enjoy having my camera on as it makes me uncomfortable and self-conscious.”*

*“I don’t like being on camera, but it’s ok when it’s with family and friends. I’m really uncomfortable talking and especially turning on my camera when talking to new people such as co-workers or classmates.”*

##### Human connection via technology

Participants reported that the use of video technology for school facilitated a sense of community, feeling supported.

*“I feel great. It’s nice to see people’s faces and to show people my face it makes me feel like I am interacting as normally as I can with others.”*

*“I didn’t mind and actually kind of preferred the extra human aspect of video/facial expressions versus just a phone call.”*

##### Disruptive to learning

Some participants reported technical issues with video technology or Wi-Fi connection disruptive to their learning (e.g., missing lectures or class material due to technical difficulties).

*“Also internet connection errors with* [digital platform] *happen often, which gets annoying because I miss class material, or the lecture gets disrupted (when the professor loses connection).”*

*“Our teachers actually tell us to not have our cameras on, which is nice. Other than that, online lectures could be good and bad. It’s bad when the teacher keeps cutting out every 5 seconds and good when the Wi-Fi doesn’t cut out and you can actually hear your teacher the whole time.”*

##### Physical complaints

Due to the extended periods of sitting and screen time, some participants reported feeling tired or drained and having frequent headaches and eye and body strain.

*“Things like bad posture and worsening eyesight may be the biggest issues for me. . . especially because I get headaches easily (have a past with concussions and too much screen time is not good for me). My classes are 8 hours in a row on Tues/Thurs, so it is a lot of back-to-back screen time.”*

*“Using technology a lot has made me more concerned for my vision, especially being exposed to high amount of blue light.”*

##### Security/privacy issues

Participants reported issues with data security as some felt that video platform service providers could use their data without their permission. Others reported privacy concerns because their living space could be seen while on camera or their image could be captured without their knowledge during on-camera sessions.

*“I hate having to be on camera so often. It makes me feel insecure and uncomfortable, especially since someone could take a picture of me and I don’t know everyone in the call, so I can’t trust them.”*

*“I don’t care about the camera but I care that my data is being harvested on a platform I’m forced to use for school. . .”*

## Discussion

This study examined youth’s experiences with attending school during the COVID-19 pandemic. A substantial minority (10%) reported not attending school in fall 2020 despite previous intentions to attend school. These participants tended to be of male gender and to have had previous mental health concerns, although these results only approached statistical significance. Participants reported both positive and negative experiences with various aspects of attending school during the pandemic, with some reporting that they were happy with the level of support they were receiving from their instructor and others reporting a lack of instructor support. Some participants also reported that their ability to adapt to remote learning improved their school experience, while others struggled to adapt. Participants had suggestions for ways the school system could better manage the remote learning system. Challenges with technology were also reported.

Interestingly, approximately 10% of participants who reported planning to attend school prior to the pandemic did not attend, and the majority of them also reported not being employed. This group appeared to consist of more boys/men, youth with less education, and youth with poorer self-rated mental health; however, associations were small and no other variables significantly differentiated this vulnerable group. It will be important for future research to examine why some students decided not to attend school and to what degree the COVID-19 pandemic influenced this decision. Participants not engaged in employment, education or training (e.g., NEET) are of particular interest, as they are at higher risk for economic and psychosocial problems (Henderson et al., 2017). Since youth are at a critical developmental period for vocational and career development, and at a period associated with mental health challenges (Bailen et al., 2019; Hawke et al., 2020; Merikangas et al., 2010), it is essential to understand the degree to which this disruption may impact youth developmental milestones or trajectory such as transition to further schooling (e.g., higher education) or entry into the workforce. This knowledge may also strengthen youth support services such as supported education and employment programs for youth (Davidson & Arim, 2019; Kutsyuruba et al., 2019).

Attending school during the COVID-19 pandemic has been difficult for many students, but there are areas of adaptation and perseverance (OECD, 2020). For youth, this is a crucial developmental period, as they are laying the foundation for their health and wellbeing that can determine health trajectories in later life, while also establishing their educational foundation leading to future careers (Matthews et al., 2019; Merikangas et al., 2010; Patton et al., 2016). In previous studies, students who attended school during the pandemic have reported poor academic performance, trouble concentrating, social isolation, depressed and suicide-related thoughts, increased workload, lack of motivation, disruptions in sleep patterns and irregular eating patterns (Rogers et al., 2021; Scott et al., 2021; Son et al., 2020). Previous findings suggest that a student’s adaptability and social support are related to their perceived experience with school during the COVID-19 pandemic (Besser et al., 2022). Those who perceived themselves as adaptable were better able to modulate their emotions and more able to adapt to the new format (Besser et al., 2022). In our sample, some participants reported challenges with self-motivation and time-management with respect to virtual learning during the pandemic. Conversely, participants who reported the ability to adapt to the new format felt more positive about their school experience, despite reporting challenges with the virtual learning. From a strengths-based lens, this is important as it likely speaks to participant’s ability to adjust to new and uncertain situations and their ability to cope and even thrive in light of new circumstances (Besser et al., 2022; Waters et al., 2020). Focusing on these strengths will be important for supporting youth in continuing to adapt to the disruptions imposed by the COVID-19 pandemic and for their own growth in future years (Waters et al., 2020). Future research should examine the implications of student adaptability or ability to self-manage on the delivery of learning content in an effort to develop early intervention strategies and other student supports such as mental health counseling.

The school system can have a powerful influence on youth development (Lloyd, 2005; Lombardi et al., 2019). It is not only where academic learning takes place, but it is also a platform for promoting wellbeing through which youth learn about the mechanisms that contribute to healthy behaviors and relationships and develop a sense of community (Patton et al., 2016; Pigaiani et al., 2020). A positive school environment can have cumulative effects for youth, including healthier behaviors, greater cognitive capacity, longer productive adult lives, better health and lower mortality among their own children, and overall greater productivity in the future workforce experience (Lloyd, 2005; Patton et al., 2016). Positive school relationships such as the relationship between youth and instructors is important, as youth connectedness to school or teachers has been positively associated with better mental health, mental health service use and wellbeing (Halladay et al., 2020; Patton et al., 2016; (Moore et al., 2018). Instructor support is also significantly related to effective online learning (Aristovnik et al., 2020; Bao, 2020; Wu & Liu, 2013) and greater academic engagement (Ye et al., 2022) and connectedness to peers is associated with improved wellbeing and mental health (Moore et al., 2018) With respect to how well schools are handling the pandemic, participants reported a lack of support from their schools and instructors. Some participants reported challenges, such as an emphasis on self-directed learning and lack of meaningful or relevant learning content, while other participants reported struggling to keep up with school deliverables and the toll this was taking on their mental health. Future research is needed to evaluate the impact of remote learning on student wellbeing and mental health, including longitudinal research to examine the long-term effects.

Studies examining the use of virtual platforms for school related activities during the COVID-19 pandemic found that extended periods of online activity was associated with higher feelings of stress, burnout and higher levels of depression (Mheidly et al., 2020) than previously experienced prior to the pandemic (Ellis et al., 2020). Similar to our own findings, participants reported feeling tired, stressed and drained. Increasing social isolation during the pandemic has been shown to predict greater mental health symptoms (Hamza et al., 2021), as psychological distress during the pandemic is significantly associated with increasing sadness, depressive symptoms, anxiety symptoms, posttraumatic stress disorder symptoms, and burdensomeness among university students without pre-existing mental health needs (Hamza et al., 2021; Son et al., 2020). In our sample, participants reported increased feelings of isolation from family and friends and the inability to meet new people while attending school during the pandemic. Some participants also reported that they appreciated connecting with peers via video technology for social interaction and a sense of community. For youth, who need social interaction and relationships with peers (Matthews et al., 2019; Pigaiani et al., 2020; Power et al., 2020; Steinberg & Morris, 2001) during this sensitive developmental period, it will be important to intervene early and leverage youth-serving support systems such as incorporating and strengthening the quality of online mental health and wellness supports for students (Toquero, 2020). Some participants also reported technical issues with video technology or Wi-Fi connection. The digital divide, limited or no access to computers and the internet, has become more evident as schools switched from in-person to remote learning during the pandemic. Largely attributed to social inequalities, the digital divide has reduced educational access to almost half of students around the world (Alvarez, 2021). As we navigate our way through a post-pandemic world, governments, policy makers, and educational institutions need to prioritize digital inclusion to ensure that every student has access to education regardless of social and economic status.

From a stakeholder-engaged perspective, we worked directly with youth members of the McCain Centre Youth Engagement Initiative (YEI) (Heffernan et al., 2017) to develop our survey questions. We shared findings with three youth team members. Our findings resonated with our youth team members and with the viewpoints they are hearing from youth across Canada. They agreed with the many challenges with attending school during the COVID-19 pandemic such as video conferencing fatigue, isolation, too much focus on self-directed learning and not enough instructor engagement and the need for tuition re-adjustment. Youth team members also identified some encouraging aspects of attending school. For example, youth enjoyed the flexibility of online learning as well as the use of video technology to keep in touch with peers. They also appreciated the importance of school social distancing measures as a means for keeping everyone safe from COVID-19. They identified that adjusting to the new learning format required a steep learning curve for youth—particularly for those who struggled with self-management. Nonetheless, they expressed that most youth were persevering and trying their best to make it work.

### Strengths and Limitations

This study reports on questions co-designed with youth, enhancing their relevance to youth experiences. Findings from this study provide timely feedback on the challenges of attending school during the pandemic and areas for improvement. This survey was conducted in Ontario, Canada. A more diverse sample collected outside of Ontario would improve generalizability. This survey was conducted electronically and thus it is likely that students without access to a computer and internet are not represented in this sample. Qualitative data were based on survey responses and not interviews. We were unable to discern the reasons participants decided to attend school, or not, during the COVID-19 pandemic. We were unable to determine the extent to which contracting COVID-19 or being quarantined may have impacted student’s mental health. Future research should examine what factors influenced youth’s decision to attend school during the COVID-19 pandemic, the role the pandemic played in their decision-making process, the extent to which this may or may not have subsequently affected their school experience, and differential experiences in secondary versus post-secondary education.

## Conclusions

With the uncertainties imposed by the COVID-19 pandemic, it is unclear how long remote learning will be required and what its impact will be on student learning. While participants appreciate the flexibility of online learning, some also reported experiencing a lack of support from their school and the need for instructor training on how to deliver virtual classes effectively. Disruption to regular school activities may negatively impact developmental milestones and education and employment trajectories for youth. Future studies should examine what factors influence student engagement with virtual learning, what strategies could improve supports for student in their long-term career development, and the longitudinal experiences of youth who may have chosen not to go back to school due to the pandemic. The benefits experienced by some youth in remote learning should be considered when planning post-COVID-19 recovery of educational and mental health supports.
